# Left Ventricular Assist Device Implantation with Concomitant Aortic Valve and Ascending Aortic Replacement

**DOI:** 10.1155/2018/9057351

**Published:** 2018-01-09

**Authors:** Katharina Huenges, Bernd Panholzer, Jochen Cremer, Assad Haneya

**Affiliations:** Department of Cardiovascular Surgery, UKSH, Kiel, Germany

## Abstract

Left ventricular assist device (LVAD) is nowadays a routine therapy for patients with advanced heart failure. We present the case of a 74-year-old male patient who was admitted to our center with terminal heart failure in dilated cardiomyopathy and ascending aortic aneurysm with aortic valve regurgitation. The LVAD implantation with simultaneous aortic valve and supracoronary ascending aortic replacement was successfully performed.

## 1. Background

Left ventricular assist device (LVAD) currently establishes a place in the treatment of end-stage heart failure as bridging therapy for patients awaiting heart transplantation or as destination therapy. Over the last decade, technical improvements in LVAD equipment, surgical implantation techniques like minimally invasive strategies, and patients' management have reduced procedure-related complications and improved the outcome and quality of life [1].

Nevertheless, additional surgery for preexisting complex cardiac pathologies in this challenging group of patients remains as one of the riskiest heart procedures with highly variable outcomes. The case of a LVAD implantation with concomitant aortic valve replacement and supracoronary ascending aortic replacement in an end-stage heart failure patient due to dilated cardiomyopathy is presented.

## 2. Case Description

A 74-year-old male patient with progressed dilative cardiomyopathy with dyspnea, peripheral edema, and chronic renal failure was evaluated for LVAD implantation as destination therapy. Despite optimization of heart failure medications, including levosimendan, the clinical status worsened (INTERMACS 3, NYHA III-IV, and NTproBNP 37276 ng/l). Preoperative echocardiography revealed a highly reduced LV function (LVEF < 20%), with still compensated RV function, an eccentric aortic valve regurgitation II°-III° with an aortic ascending aneurysm of 5.6 cm diameter (LVEDD 80 mm, MAPSE 3 mm, PASP 50 mmHg + CVP, TAPSE 13 mm, and PHT 400 ms). Computed tomography (CT) confirmed those findings. Besides his cardiac and renal diseases, no contraindications were found for LVAD implantation, and the patient was prepared for elective surgery.

**Figure 1:**
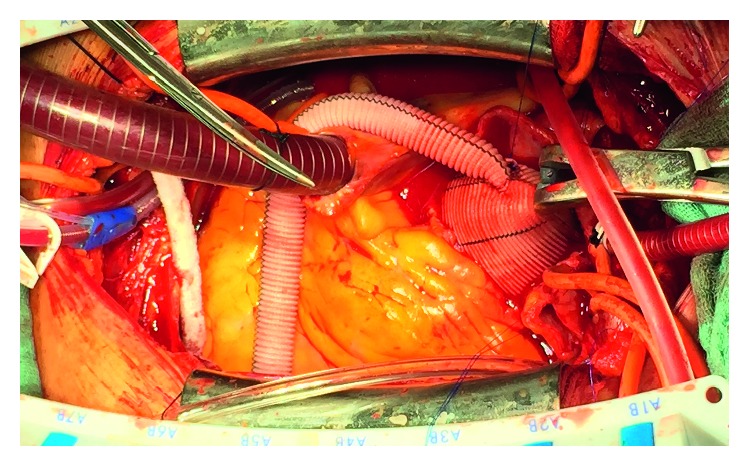
Intraoperative situs.

Median sternotomy was chosen as a surgical approach, and cannulation for cardiopulmonary bypass (CPB) was routinely performed with aortic cannulation and venous cannula in the right atrium. Myocardial protection was obtained in all cases with retrograde delivery of cold blood cardioplegic solution. The distal anastomosis of the supracoronary ascending aorta replacement (34 mm AlboGraft, LeMaitre Vascular) was performed in deep hypothermic circulatory arrest (24°C). Near-infrared spectroscopy (NIRS) is also used to observe the cerebral oxygenation. By completing the anastomosis, CPB was restarted with the perfusion cannula directly inserted into the graft. During rewarming, the LVAD (HVAD, HeartWare, Medtronic) was implanted. After placement of multiple buttressed sutures, an apical access to the left ventricle was created, and the apex cannula was inserted, fixed, and deaired. The aortic valve replacement (29 mm Hancock II, Medtronic) was followed by the proximal ascending aortic anastomosis. The outflow graft was anastomosed in the ascending aortic graft with a graft-in-graft end-to-side anastomosis with a continuous suture (4-0 Prolene^®^, Ethicon) (Figure 1). The LVAD pump was started, and the patient could be weaned off the CPB. After aortic graft inclusion and extended bleeding control, the chest was closed. The skin-to-skin time was 290 min, with an acceptable CPB time of 190 min. Under low catecholamine as well as inotropic support and NO ventilation, the patient was transferred to our intensive care unit (ICU). The direct postoperative course was impaired by high drainage loss requiring rethoracotomy, without detection of a surgical bleeding. No relevant drainage loss occurred in the following postoperative days. Postoperatively, no relevant pericardial effusion or hematoma was detected in TTE with normal aortic prosthesis function. After 12 hours without bleeding, we started anticoagulation with heparin (PTT 50–60 sec). Long-term anticoagulation consisted of phenprocoumon, together with a platelet aggregation inhibitor at a low dosage (100 mg acetylsalicylic acid daily).

The NO ventilation could be weaned off with no detectable signs of right ventricular decompensation. The VAD pump speed was adjusted to achieve an optimal LV unloading and to facilitate aortic valve opening in a 2 : 1 ratio, according to echocardiography. The patient was extubated at the first operative day. After initial quick recovery and extubation, the patient developed a severe additional pneumonia requiring reintubation. The patient also had multiple organ failure. Unfortunately, the prognosis was poor and further therapy was limited, and he died three weeks after surgery.

## 3. Conclusion

Preexisting cardiac pathologies are common in patients with end-stage heart failure undergoing LVAD implantation, and cardiac surgeons are nowadays faced with treating an increasing number of these challenging patient groups. Morgan et al. demonstrated that concomitant cardiac procedures like aortic or tricuspid valve surgery during LVAD implantation are not assumed with higher procedural risks [2]. Maltais et al. reported about similar findings but emphasized the patient-to-patient-wise decision-making with focus on age, preoperative renal function, and the complexity of planned surgery [3].

In our case, the patient was elder with chronic renal dysfunction (KDOQI/KDIGO G3b). The ascending aortic replacement with deep hypothermic circulatory cardiac arrest and additional aortic valve replacement during LVAD implantation was extensively discussed. Ascending aortic replacement combined with simultaneous VAD implantation has not been reported so far. But since LVAD implantations in patients after ascending aorta surgery have been reported [4], the feasibility of simultaneous VAD implantation with ascending aortic surgery might be assumable.

The order of the surgical steps, distal ascending aortic anastomosis, LVAD implantation, aortic valve replacement, proximal ascending aortic anastomosis, and the outflow-graft in graft anastomosis at last proved to be a good concept, facilitating surgical overview.

In conclusion, our experience suggests that an additional complex cardiac procedure during LVAD implantation is a safe alternative with encouraging results.
